# Prefrontal-posterior coupling while observing the suffering of other people, and the development of intrusive memories

**DOI:** 10.1111/psyp.12197

**Published:** 2014-02-24

**Authors:** Eva M Reiser, Elisabeth M Weiss, Günter Schulter, Emily A Holmes, Andreas Fink, Ilona Papousek

**Affiliations:** a Department of Psychology, Biological Psychology Unit, University of GrazGraz, Austria; b MRC Cognition and Brain Sciences UnitCambridge, UK

**Keywords:** Intrusions, Implicit memory, EEG coherence, Intrahemispheric communication, Top-down modulation

## Abstract

Witnessing the suffering of others, for instance, in hospital emergency rooms but also through televised images in news or reality programs, may be associated with the occurrence of later intrusive memories. The factors contributing to why some people develop intrusive memories and others do not are still poorly understood. *N* = 121 healthy women were exposed to film scenes showing the suffering of dying, severely injured, and mourning people while their EEG was recorded. Individuals showing greater decreases of functional coupling between prefrontal and posterior cortices (greater decreases of EEG beta coherences) reported more intrusive memories of the witnessed events. This was shown for intrusions in the short term (immediately after viewing the film) as well as in the medium term (intrusive memories over 1 week). The findings illuminate brain mechanisms involved in the encoding of information in ways that make intrusive memories more likely.

Experiencing horrifying events such as a violent assault or a severe road traffic accident oneself but also witnessing death and suffering of others has been associated with the occurrence of later intrusive memories. They are, for instance, frequently reported by medical and paramedical personnel who work in hospital emergency rooms, and several studies have suggested that even observing the suffering of other people through televised images can lead to the development of intrusive memories (Breslau, Bohnert, & Koenen, 2010; Durham, McCammon, & Alison, 1985; Schlenger et al., 2002; Schuster et al., 2001). Indeed, the recent Diagnostic and Statistical Manual of Mental Disorders, 5th ed. (American Psychiatric Association, 2013) has amended the criteria for posttraumatic stress disorder (PTSD) to include the viewing of traumatic film footage in the line of work as an index traumatic event. Intrusive memories, or intrusions, are unwanted spontaneously occurring recollections of past events. They are a symptom of various psychopathologies such as PTSD as well as depression, obsessive-compulsive disorders, and other anxiety disorders (Brewin, Gregory, Lipton, & Burgess, 2010; Holmes & Hackmann, 2004), but also occur in everyday life and can vary on a continuum from only mildly burdensome forms to very distressing forms of flashbacks (Horowitz, 1975; Krans, Näring, Becker, & Holmes, 2009). In contrast to deliberately retrievable memories, which are verbally reportable with voluntarily recalled explicit memory content, intrusive memories are thought to arise from a more implicit or involuntary memory (Brewin, Dalgleish, & Joseph, 1996; Ehlers & Clark, 2000; Holmes, Brewin, & Hennessy, 2004).

To date, little evidence exists as to why certain people develop intrusive memories and others do not. It has been argued that individual differences in the processing of sensory information during a distressing event may lead to differences in the proportion of explicit and implicit memories of the event, which may be crucial for the development of intrusive memories (Holmes et al., 2004). There is some evidence suggesting that these individual differences in peritraumatic processing may be related to distinct variations of the functional coupling between prefrontal and more posterior cortical areas while experiencing or witnessing a distressing event.

The acquisition of explicit memories is accompanied by activity in large-scale cortical networks, including an increase of functional coupling between prefrontal and more posterior, perception-related cortical areas (McIntosh, Rajah, & Lobaugh, 1999; Rose, Haider, & Büchel, 2010; Wessel, Haider, & Rose, 2012). A recent model concerning conscious and nonconscious processing, based on the global workspace model of Baars (1988, 1997, 2002), suggested that sensory information only enters awareness and becomes verbally reportable if bottom-up stimulus strength and top-down attentional amplification is mobilized to a sufficient magnitude (Dehaene & Changeux, 2011; Dehaene, Changeux, Naccache, Sackur, & Sergent, 2006). Specifically, it was proposed that sensory input leads to cortical feedforward activation ensuing from early extrastriate areas. Since this bottom-up activation declines depending on strength and feedforward duration, it is not thought to be sufficient for conscious processing. Orientation of attention to the incoming sensory information results in top-down amplification of the activation and ignition of a large-scale prefrontal-posterior network. Through prefrontal feedback control achieved by long-distance coupling between prefrontal cortical regions and posterior sensory areas, the activation is maintained to a sufficient degree to enable evaluation of the sensory information and its encoding in long-term memory. Sensory input that is processed in this manner becomes explicit memory content, whereby input that does not reach the threshold for conscious processing because of weak bottom-up stimulus strength or insufficient top-down modulation by the prefrontal cortex will be stored as implicit memory. In line with the model by Dehaene and colleagues (Dehaene & Changeux, 2011; Dehaene et al., 2006), it has been proposed that if insufficient attention is directed towards sensory information, this information will be encoded as implicit memory. Severe emotional distress, for example, during experiencing or witnessing horrifying events, narrows the attentional focus and therefore limits consciousness processing and explicit memory encoding. Information that is encoded as implicit memory may later “pop up” as intrusions (Brewin, 2001; Brewin et al., 1996; Cohen, Cavanagh, Chun, & Nakayama, 2012; Holmes et al., 2004). Taken together, there is evidence that the functional connectivity between prefrontal and more posterior cortical regions during the processing of incoming sensory information may be related to the formation of implicit memories and, consequently, to the development of intrusions. We seek to test this possibility in the current study.

Intrusive memories are also more affect laden than explicit memories, usually implicating the feelings that the distressing event had initially evoked (Brewin, 2001; Ehlers & Clark, 2000). In the context of affective processing, the prefrontal cortex receives highly processed sensory information and in turn exerts feedback control on posterior association cortices in order to further modulate representations of affectively relevant information (Miskovic & Schmidt, 2010; Rudrauf et al., 2008; see also Vuilleumier & Driver, 2007). Recently, it was demonstrated that an increase in functional connectivity between prefrontal and more posterior cortical areas, as is also expected with explicit memory encoding, attenuates the emotional impact that an event has on the individual (Papousek et al., 2013; Reiser et al., 2012). It has been proposed that loosening of modulatory control over emotionally laden sensory input, by further opening the perceptual gate, leaves individuals relatively unprotected from becoming affected by the perception of emotional information, and that these modulatory processes may not only influence the perception and experience of affect but also the encoding and later recall of emotional content (Miskovic & Schmidt, 2010; Papousek et al., 2013; Reiser et al., 2012). Therefore, reduced top-down modulation of distressing sensory information, indicated by reduced prefrontal-posterior coupling, may also result in more affect-laden memories (see also Brewin, 2001). These relationships further support the notion that individual differences in prefrontal-posterior coupling during the perception of distressing information may be a strong candidate for explaining variability in the development of intrusions.

Coupling and de-coupling of prefrontal and posterior cortical regions in the context of relevant modulatory processes can be assessed by electroencephalogram (EEG) coherence measures (Miskovic & Schmidt, 2010; Papousek et al., 2013; Reiser et al., 2012; Wessel et al., 2012). Increases of EEG coherences are considered to indicate increased connectivity and functional communication between two neuronal populations whereas decreases indicate a decline in functional communication (Fries, 2005; Srinivasan, Winter, Ding, & Nunez, 2007).

In the current study, participants were exposed to film scenes showing dying, severely injured, and mourning people. The film content was similar to that witnessed by television viewers watching programs such as news coverage of road traffic accidents, or programs about the police or ambulance service work. We hypothesized that those individuals showing relatively greater decreases of functional coupling of prefrontal and temporoparietal cortical regions while viewing the film would report more intrusive memories of the film content. Particularly, changes of prefrontal-posterior EEG coherences in the right hemisphere were expected to be predictive of intrusive memories. Previous research on the relevance of prefrontal-posterior coupling to explicit memory encoding as well as to affective processing indicated greater importance of the right than of the left hemisphere in relevant modulatory processes (Papousek et al., 2013; Reiser et al., 2012; Wessel et al., 2012).

The occurrence of intrusive memories was assessed in the short term (directly after viewing the film) and in the medium term (during the subsequent week), in order to examine whether intrusions persisted at least for some time.

## Method

### Participants

One hundred and twenty-four right-handed female university students completed the experiment with all required data. A female-only sample was chosen because previous research indicated that women are more reactive in a lab situation to negative emotional stimulation than men, particularly if the stimulation is threatening or traumatic (Whittle, Yücel, Yap, & Allen, 2011). Only women who confirmed that they had not had real traumatic experiences related to car crashes, surgery, or death of a close person within the past 12 months and did not have a neuropsychiatric disease were admitted to the study. Individuals who reported using psychoactive medication or whose scores on the Beck Depression Inventory exceeded the threshold for severe depressive symptoms were excluded from the study (*n* = 3). The final sample comprised 121 women aged 18 to 59 years (*M* = 22.5, *SD* = 4.9). Handedness was assessed by a standardized handedness test (performance test; Papousek & Schulter, 1999; Steingrüber & Lienert, 1971). Participants were requested to come to the study well rested and to refrain from alcohol for 12 h, and from coffee and other stimulating beverages for 2 h, prior to their lab appointment. The study was performed in accordance with the 1964 Declaration of Helsinki and the American Psychological Association's Ethics Code and was approved by the local ethics committee. Participants gave their written and informed consent to participate in the study.

### Stimulus Material

Participants were exposed to a film (approximately 10 min in length) containing 11 clips that have been used in previous studies as an experimental analogue of psychological trauma. They have been shown to induce significant levels of emotional distress (Holmes & Bourne, 2008; Holmes, James, Coode-Bate, & Deeprose, 2009; Holmes, James, Kilford, & Deeprose, 2010). EEG was recorded during the last 5 min of the film, during which there were five clips depicting several car accidents and a rampaging elephant injuring people at a circus. These clips included graphic scenes of severely injured, dying, and mourning people. The film was displayed on a 21″ computer monitor viewed at 100 cm and was presented without sound, so that the stimulation was dominated by visual information for all participants. The neutral visual display, used for obtaining the reference data, showed a green circle (diameter 90 mm) at the center of the screen.

### Self-Report Measures

#### Intrusive memories assessed in the short term

Two minutes after viewing the film, participants were asked to rate the frequency of involuntarily appearing images from the film in their mind's eye that occurred during the 2-min rest period following the film (4-point rating scale ranging from 0 (*not at all*) to 3 (*six times or more*); *M* = 1.2, *SD* = 1.0). Participants indicated their judgment via a click with the mouse.

#### Intrusive memories assessed in the medium term

The intrusion subscale of the Impact of Event scale (IES-R; German adaptation, Maercker & Schützwohl, 1998) was used, adapted to refer to the film as the index event. It consists of seven items referring to the occurrence of intrusive thoughts, nightmares, intrusive feelings, and imagery associated with the event over the week. Scores have a potential range from 0 to 35 (in the present sample, *M* = 6.4, *SD* = 4.8; internal consistency reliability α = .78). Additionally, participants were asked to keep a pen and paper daily diary in which they recorded spontaneous intrusive images of the film over a period of 1 week and also described each intrusion's content for verification that they indeed matched the film content (cf. Bourne, Mackay, & Holmes, 2013; Holmes & Bourne, 2008; Holmes et al., 2004, 2009, 2010). Only entries that passed this verification were included in the analysis. The number of diary entries qualifying as intrusive images ranged from 0 to 11 (*M* = 1.7, *SD* = 2.0).

#### Depression. 

Depressed mood was assessed using the Center for Epidemiologic Studies Depression Scale (CES-D; German adaptation, Hautzinger & Bailer, 1993). It is comprised of 20 items referring to mood and attributions over the past week and is designed for measuring subclinical depressive experiences in the general population (Wood, Taylor, & Joseph, 2010). Scores have a potential range from 0 to 60 (in the present sample, *M* = 11.3, *SD* = 6.0, α = .78).

#### Subjective impact of the stimulus

Participants rated the degree to which the film had affected them on a 10-cm horizontal visual analogue scale. The responses were scored in millimetres from 0 (*not at all*) to 100 (*extremely*); *M* = 73.1, *SD* = 22.3.

### EEG Recording and Quantification

The EEG was recorded from 19 channels according to the International 10–20 system, using a Brainvision BrainAmp Research Amplifier (Brain Products; sampling rate 500 Hz, resolution 0.1 μV) and a stretchable electrode cap, and was rereferenced offline to a mathematically averaged ears reference (Essl & Rappelsberger, 1998; Hagemann, 2004). Impedance was kept below 5 kΩ for all electrodes. Horizontal and vertical electrooculogram (EOG) measures were obtained for identification of ocular artifacts. All data were inspected visually, in order to eliminate intervals in which ocular or muscle artifacts occurred. All participants had at least 30 s of artifact-free data in each of the recording periods and in each of the electrode positions of interest. The mean numbers of artifact-free epochs were *M* = 116.8 (*SD* = 44.8) for the baseline recording and *M* = 215.0 (*SD* = 109.1) for the film recording. Artifact-free EEG data were submitted to fast Fourier analysis using a Hanning window (epoch length 1 s, overlapping 50%; low-cut filter 0.016 Hz). Spectral coherence (Fisher's *z* transformed) was obtained in the beta band (13–30 Hz) using the quotient of the cross spectrum (CS) and the auto spectra according to the following equation: Coh(c_1_,c_2_)(f) = |CS(c_1_,c_2_)(f)|^2^ / (|CS(c_1_,c_1_)(f)| |CS(c_2_,c_2_)(f)|), with CS(c_1_,c_2_)(f) = Σc_1,__i_ (f) c_2,__i_ (f). Coh(c_1_,c_2_)(f) denotes the coherence at frequency f between electrodes 1 and 2, which can vary between 0 and 1.

We focused on EEG coherence in the beta frequency range (13–30 Hz), because previous research indicated that state-dependent changes of cortical connectivity during affective processing occurred primarily in the beta frequency range (Aftanas, Lotova, Koshkarov, & Popov, 1998; Miskovic & Schmidt, 2010; Papousek et al., 2013; Reiser et al., 2012). In addition, studies emphasized the particular relevance of connectivity changes in higher frequency ranges to explicit memory encoding (Rose et al., 2010; Wessel et al., 2012). Research also suggested a particular importance of beta-band oscillations for mediating long distance coupling in general (Gross et al., 2004; Kopell, Ermentrout, Whittington, & Traub, 2000; Schnitzler & Gross, 2005).

Following previous relevant research (Miskovic & Schmidt, 2010; Papousek et al., 2013; Reiser et al., 2012), coherence pairs were grouped into anatomically valid clusters corresponding to the left and right, prefrontal and posterior association cortex regions. Coherence scores of nine electrode pairs each were averaged to summarize interaction within the left and the right hemisphere, respectively (left: Fp1-T7, Fp1-P3, Fp1-P7, F3-T7, F3-P3, F3-P7, F7-T7, F7-P3, F7-P7; right: Fp2-T8, Fp2-P4, Fp2-P8, F4-T8, F4-P4, F4-P8, F8-T8, F8-P4, F8-P8). By using these clusters, we avoided a hardly manageable inflation of the number of statistical tests. The selection of the posterior electrodes was in accordance with evidence of involvement of the posterior part of the temporal lobe and the inferior parietal lobe in the visual perception of socially relevant information (Decety & Sommerville, 2003).

### Procedure

**Figure 1:**
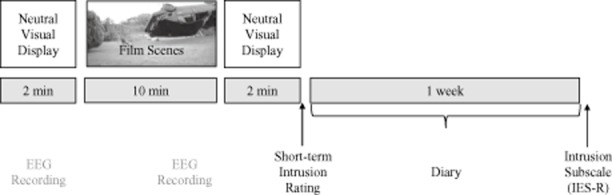
Study design overview. Participants were exposed to a film containing several scenes that had been used in previous studies as an experimental analogue of psychological trauma. EEG was recorded during the last 5 min of the film and during the 2-min reference period preceding the film. Short-term impact in terms of intrusive images occurring during the 2 min following the film was assessed immediately afterwards. Medium-term impact was assessed over a period of 1 week with a daily pen and paper diary in which participants recorded intrusive images of the film and using the intrusion subscale of the Impact of Event Scale (IES-R), administered 1 week after the initial test session.

After completing the handedness test and the CES-D, participants were seated in an acoustically and electrically shielded examination chamber, and electrodes were attached. Participants were then instructed that, after a short recording period during which they should watch the green circle on the screen (2 min), they would see a film to which they should direct their whole attention. They were asked to view the film as if they were really there, like a bystander at the scene of the events and to not close their eyes or look away. The film would be followed by another 2-min rest period. Subsequently, the short-term intrusion rating and the rating to what degree the film had affected the participant appeared on the screen, which the participants completed using the computer mouse. The technical equipment and the experimenter were located outside the EEG chamber. The participants were continuously monitored by a camera. Participants then were instructed to keep a daily diary for 1 week, in which they recorded their intrusions of the film scenes. On return to the laboratory 1 week after the EEG recording, participants delivered the diary and completed the IES-R intrusion scale.^1^ See Figure 1 for an overview of the study design.

### Statistical Analysis

Following previous relevant research (Papousek et al., 2013; Reiser et al., 2012), linear regressions were conducted using the EEG beta coherence during the reference period preceding the film to predict the coherence during viewing the film, in order to calculate residualized change scores. These were used as an index of state-dependent decreases or increases of intrahemispheric coherence in response to observing the suffering of other people. This was done to ensure that the analyzed residual variability was due to the experimental manipulation, and not to individual differences in baseline levels, and to control for measurement error inherent in the use of repeated measures of the same kind (e.g., Linden, Earle, Gerin, & Christenfeld, 1997; Steketee & Chambless, 1992). In the following, the abbreviation “Δcoh” will be used for these change-of-coherence scores. Negative scores indicate a decrease in prefrontal-posterior coherence; positive scores indicate an increase.

To evaluate whether interindividual differences in the coherence changes during observing the suffering of other people may predict the later occurrence of intrusive memories, multiple regression analyses were conducted, with one of the indicators of intrusive memories (short-term rating, IES-R intrusion scale, or diary) as the dependent variable and the change-of-coherence score (Δcoh) and depression as predictors. Depression was controlled because there is evidence that depressed mood can affect the likelihood of intrusive memories (Brewin, Reynolds, & Tata, 1999; Brewin, Watson, McCarthy, Hyman, & Dayson, 1998). Entering Δcoh and depression simultaneously in the model allowed us to determine the unique contribution of each. A significant semipartial correlation (*sr*) for Δcoh indicates that Δcoh explained a significant amount of variance of intrusive memories, independently of depression. *Sr*^2^ indicates the amount of unique variance and thus the size of the unique effect of Δcoh. As previous research suggested lateralized effects of prefrontal-posterior coupling on implicit memory encoding (Rose et al., 2010; Wessel et al., 2012) and affective processing (Papousek et al., 2013; Reiser et al., 2012; Schellberg, Besthorn, Klos, & Gasser, 1990), coherence changes were analyzed separately for the left and the right hemisphere.

## Results

### Intrusive Memories Occurring Immediately After Observing the Suffering of Other People

Δcoh in the right hemisphere in response to the film predicted the incidence of intrusive images in the 2-min rest period following the film, *F*(2,118) = 4.2, *p* < .05; β = −.21, *p* < .05. Independently from depressed mood, a stronger decrease of prefrontal-posterior coherence during watching the film was associated with a higher number of intrusive images. Depressed mood also predicted the number of intrusive images, with higher depression scores being related to more intrusive images (β = .19, *p* < .05). The analogous analysis with coherence changes in the left hemisphere yielded no significant results, *F*(2,118) = 2.0, *p* = .15; Δcoh: β = −.09, *p* = .33. See Table 1 for a summary of the semipartial correlations.^2^

**Table 1:** Effects of Changes of Prefrontal-Posterior EEG Coherence During the Observation of Other People's Suffering on the Occurrence of Intrusive Memories

Intrusive memories	Depression	Δcoh Right hemisphere	Δcoh Left hemisphere
Short-term (rating)	*r* = .16	*sr* = −.20*	*sr* = −.09
Medium-term (IES-R)	*r* = .30**	*sr* = −.22**	*sr =* −.10
Medium-term (diary)	*r* = −.11	*sr* = −.04	*sr* = −.06
Depression	−	*r* = .19*	*r* = .12

*Note*. The table shows zero-order correlations (*r*) of depression with intrusive memories and coherence changes, and semipartial correlations (controlling for depression, *sr*) of Δcoh in the right and left hemisphere with intrusive memories. Negative coherence scores indicate a decrease in prefrontal to posterior coupling. The short-term rating captured intrusions occurring immediately after viewing the film. The medium-term measures captured the occurrence of film-related intrusive memories over the week.

* *p* < .05.

** *p* < .01.

### Intrusive Memories Occurring During the Subsequent Week

**Figure 2:**
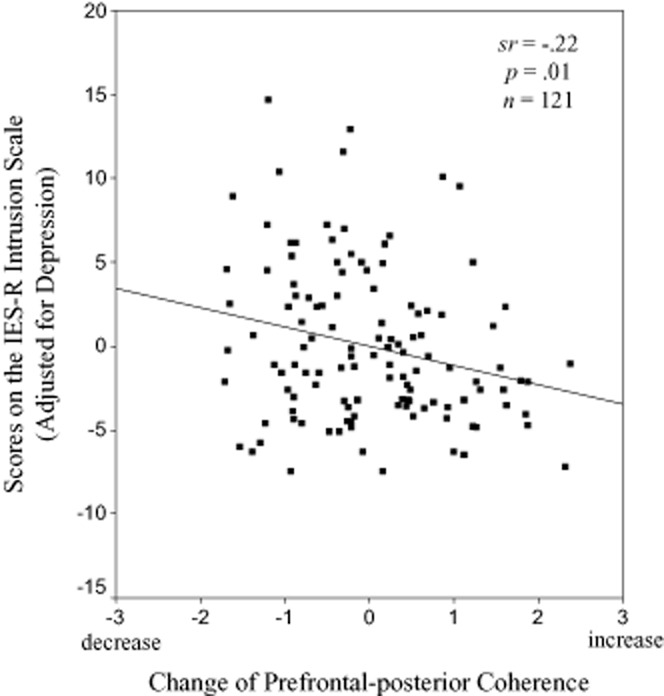
Prediction of film-related intrusive memories over the week by changes of prefrontal-posterior EEG coherence while viewing the distressing film. Coherence changes in the right hemisphere (beta frequency range) relative to reference recording preceding the film (standardized residuals, Δcoh; see Statistical Analysis section).

The analysis revealed an association between Δcoh in the right hemisphere and the participants' scores on the IES-R intrusion scale, *F*(2,118) = 9.4, *p* < .001; β = −.23, *p* < .01. Independently from depressed mood, a stronger decrease of prefrontal-posterior coherence during watching other people suffer was associated with higher scores on the IES-R intrusion scale, indicating a higher incidence of film-related intrusive memories over the week. Depressed mood was also related to higher IES-R intrusion scores (β = .34, *p* < .001). Δcoh in the left hemisphere did not predict intrusive memories reported in the IES-R, *F*(2,118) = 6.4, *p* < .005; Δcoh: β = −.10, *p* = .25; depression: β = .31, p < .001. Semipartial correlations for Δcoh are shown in Table 1. Δcoh did not predict the frequency of intrusive memories reported in the diary (right hemisphere: *F*(2,118) = 0.7, *p* = .48; Δcoh: β = −.04, *p* = .70; left hemisphere: *F*(2,118) = 0.8, *p* = .42; Δcoh: β = −.06, *p* = .52).^3^ Figure 2 shows the scatter plot of the correlation between changes of prefrontal-posterior EEG coherence while viewing the distressing film (Δcoh, right hemisphere) and film-related intrusive memories over the week (IES-R intrusion scale).

### Supplemental Analyses

To exclude the possibility that the coherence changes might have been influenced by changes in enhanced myogenic activity, we calculated correlations between the Δcohs and the respective changes of the spectral power in the range of 65–75 Hz, which is presumed to be exclusively myogenic in origin (averaged across the used electrode positions). These correlations were small and not significant (right hemisphere: *r* = −.12, *p* = .21; left hemisphere: *r* = −.11, *p* = .24). Repeating the main statistical analysis with additionally entering the changes in the 65–75 Hz frequency range into the regression models did not change the initial findings (Δcoh right hemisphere: intrusion rating β = −.19, *p* < .05; IES-R intrusion scale β = −.24, *p* < .01; diary β = −.03, *p* = .80).

To exclude the possibility that differences in the length of the recordings might have affected the results, correlations between Δcoh and the difference of used (i.e., artifact-free) epochs in the reference recording and the film recording were calculated. Correlations were *r* = −.03 (*p* = .76) for the right hemisphere and *r* = −.12 (*p* = .21) for the left hemisphere. The difference of used epochs in the two recording periods did not correlate with any of the dependent variables (short-term intrusion rating: *r* = −.07, *p* = .47; IES-R intrusion scale: *r* = −.04, *p* = .65; diary: *r* = .06, *p* = .55). Repeating the main statistical analysis with additionally entering the difference of the number of artifact-free epochs into the regression models did not change the initial findings (Δcoh right hemisphere: intrusion rating β = −.21, *p* < .05; IES-R intrusion scale β = −.23, *p* < .01; diary β = −.04, *p* = .70).

To further evaluate the specificity of the findings, we also tested potential effects of diagonal EEG coherences between the left prefrontal and right posterior clusters, and between the right prefrontal and left posterior clusters on the occurrence of intrusive memories, using linear regression analyses analogous to those in the main analysis. These coherences did not predict any of the dependent variables (all *sr*s ≤ .10), rendering the influence of a strong source affecting the signals at anterior as well as posterior electrodes and thereby producing spurious coherences between prefrontal and posterior sites of one hemisphere unlikely.

**Figure 3:**
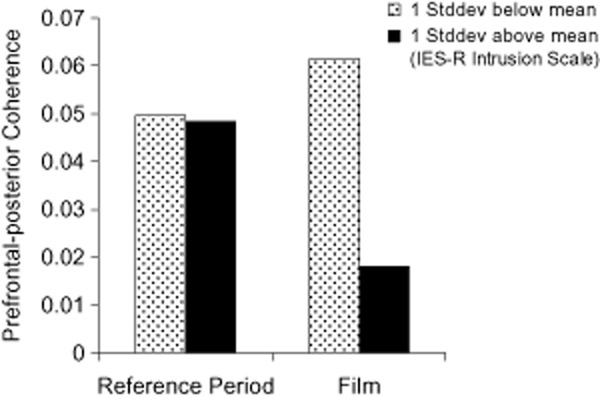
Prefrontal-posterior EEG coherence (raw scores) during the neutral reference period and the film depicting the suffering of other people. The figure shows the estimated EEG coherences in the beta frequency band (right hemisphere) in participants scoring one standard deviation above and one standard deviation below the sample mean on the IES-R intrusion scale, indicating film-related intrusive memories over the subsequent week.

**Figure 4:**
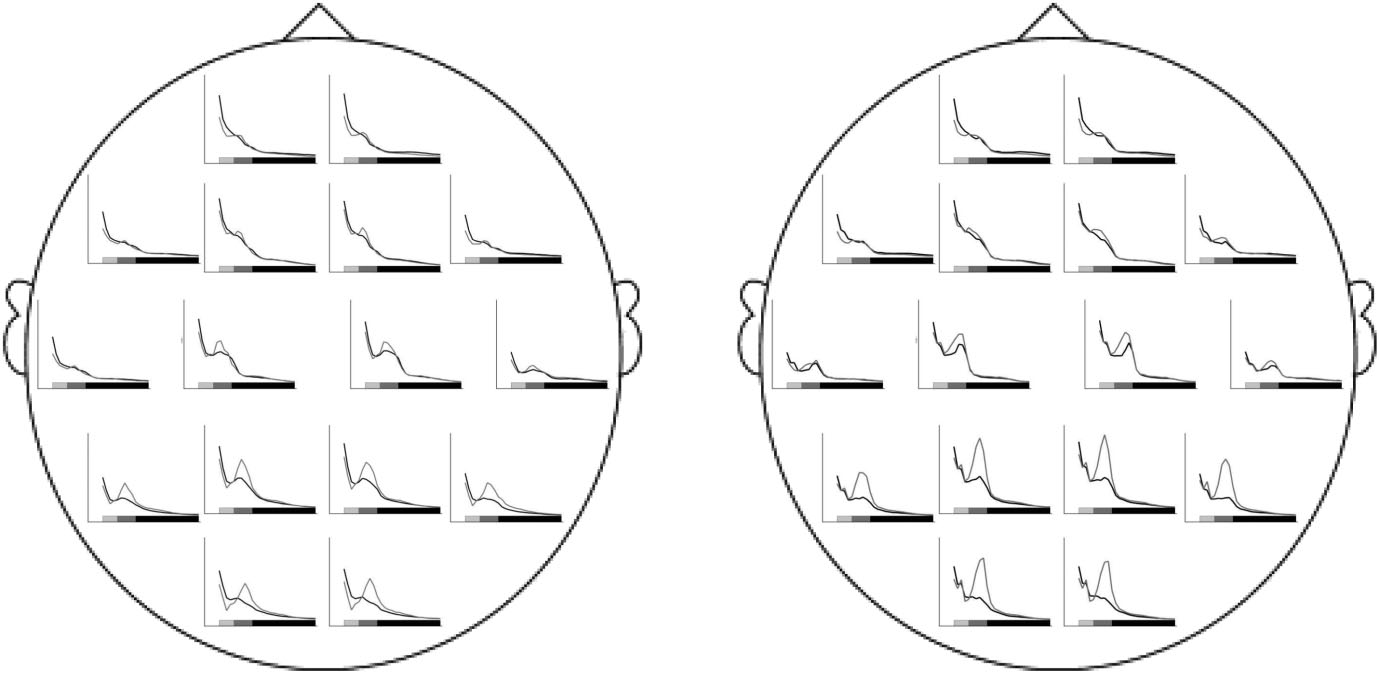
EEG power spectra during the neutral reference period and the film depicting the suffering of other people. The figure shows estimated EEG power spectra for each electrode during the neutral reference period (gray line) and during the film (black line) in participants scoring one standard deviation below (left) and one standard deviation above (right) the sample mean on the IES-R intrusion scale, indicating film-related intrusive memories over the subsequent week. The bars below the spectra mark the EEG frequency ranges: theta (4–7 Hz): light gray bar, alpha (8–12 Hz): dark gray bar, beta (13–30 Hz): black bar.

On average, EEG beta coherence decreased from the reference period to viewing the film (right hemisphere: *t*(120) = 7.1, *p* < .001; *M* = 0.049, *SD* = 0.019; *M* = 0.040, *SD* = 0.017; left hemisphere: *t*(120) = 8.0, *p* < .001; *M* = 0.051, *SD* = 0.022; *M* = 0.039, *SD* = 0.017). To illustrate the changes of prefrontal-posterior EEG coherence while observing the suffering of other people, prefrontal-posterior EEG coherences (beta frequency band, right hemisphere) during the reference period preceding the film and during viewing the film were calculated for participants scoring one standard deviation above and one standard deviation below the sample mean on the IES-R intrusion scale using linear regression (Figure 3). Figure 3 illustrates that prefrontal-posterior coherence decreased during watching the film in individuals with high scores on the IES-R intrusion scale, whereas it did not decrease in indiviuals who reported few film-related intrusions over the week. A highly similar pattern was observed relating to the short-term intrusion rating immediately following the film. In addition, we show a descriptive illustration of the EEG power spectra during the neutral reference period and during the stressful film, calculated for participants scoring one standard deviation above and one standard deviation below the sample mean on the IES-R intrusion scale using linear regression (Figure 4).

Correlations between Δcoh and depression were *r* = .19, *p* < .05 (right hemisphere) and *r* = .12, *p* = .21 (left hemisphere). Intercorrelations among the dependent variables were *r* = .30, *p* = .001 (short-term rating–IES-R); *r* = .19, *p* < .05 (rating–diary); *r* = .37, *p* < .001 (IES-R–diary).

The correlations between the retrospective rating to which degree the participants felt affected by the film with the intrusion rating briefly after viewing the film and the IES-R intrusion scale were *r* = .30 (*p* < .001) and *r* = .37 (*p* < .001), respectively. No significant correlation was observed between the rating and the number of intrusive memories reported in the diary (*r* = .15, *p* = .10). Additionally entering the rating in the regression analyses did not change the statistical results for Δcoh (significant results remained significant and nonsignificant results remained nonsignificant), indicating that the effect of the EEG coherence changes on intrusive memories was not explained by the degree to which the participants felt affected by the film.

**Figure 5:**
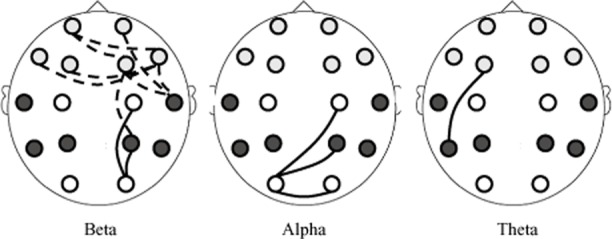
Descriptive illustration of correlations (*sr* > .20) between coherence changes of single electrode pairs and the occurrence of film-related intrusive memories over the week. Coherence changes relative to reference recording preceding the film (standardized residuals). Dotted lines denote negative correlations (linking decreased coherence during viewing the distressing film with higher scores on the IES-R intrusion scale), solid lines denote positive correlations (linking increased coherence during viewing the film with higher scores on the IES-R intrusion scale). Light shaded and dark shaded dots represent the electrodes used for the prefrontal and posterior clusters, respectively (see section EEG Recording and Quantification). In line with the statistical analysis of the aggregated prefrontal-posterior coherences, the illustration shows semipartial correlations (*sr*) controlling for depression. The illustration is for descriptive purposes only.

For descriptive purposes, a topographic illustration of the highest correlations (*sr* > .20) between coherence changes of single electrode pairs and film-related intrusive memories over the week (IES-R intrusion scale) is given in Figure 5. As the present study follows a strictly hypothesis-driven approach, statistically evaluating theoretically motivated relations of only a few specific (aggregated) coherence data (Δcoh) to the variables referring to intrusive memories, no inferences are to be drawn from this purely descriptive illustration.

## Discussion

The present study demonstrated that individuals showing greater decreases of prefrontal-posterior EEG coherences while observing the suffering of other people reported more intrusive memories of the witnessed events. This was shown for intrusions in the short term (immediately after viewing the film) as well as in the medium term, that is, for the occurrence of intrusive memories over one week, indicating that the effects persisted at least for some time. The results are in line with the notion that individual differences in information processing directly while experiencing or witnessing a distressing event (i.e., peritraumatically) are related to the development of later intrusive memories of that event (Bourne et al, 2013; Holmes et al., 2004). In addition, the present findings provide first evidence for the relevance of individual differences in changes of prefontal-posterior coupling in this particular context.

As intrusive memories are considered at least in part to arise from implicit memories, it was argued that encoding sensory information in explicit memory would be protective against the occurrence of intrusive memories (Holmes et al., 2004). Cognitive research has shown that the transfer of sensory information to explicit memory is accompanied by the activation of a prefrontal-posterior cortical network, with prefrontal cortex exerting feedback control on more posterior cortices and thus on the representations of sensory input (Dehaene & Changeux, 2011; Dehaene et al., 2006; McIntosh et al., 1999; Wessel et al., 2012). There is also evidence that abnormal neural communication plays an important role in several brain disorders where consciousness and memory processes are impaired (Dehaene & Changeux, 2011; Uhlhaas & Singer, 2006).

In affective research, similar processes are assumed in the context of processing social-emotional information. Bottom-up processing of social-emotional information, which is automatically activated by perceptual input, is supposed to be modulated in a top-down fashion through an executive control component implemented in the prefrontal cortex (Decety & Moriguchi, 2007). The relevance of these proposed interactive processes has been supported by studies using magnetic resonance imaging methods as well as by studies using EEG coherences, which also demonstrated lesser emotional impact of social-emotional information when prefrontal-posterior communication during the stimulation was strong (Diekhof et al., 2011; Papousek et al., 2013; Reiser et al., 2012). The present results add to these findings, suggesting that, independently from the degree to which one immediately feels affected by the event, state-dependent changes of prefrontal-posterior coupling during distressing events influence the encoding and later recall of such events (see also Miskovic & Schmidt, 2010). Greater increases of prefrontal-posterior EEG coherences during the processing of negative social-emotional information have also been linked to lower scores in the propensity to ruminate (Reiser et al., 2012), a personality trait that is related to cognitive as well as emotional processes associated with depression (Joormann, 2006; Joormann & Gotlib, 2008; Koster, DeLissnyder, Derakshan & DeRaedt, 2011).

Intrusive memories are automatically triggered by external or internal triggers that may be only remotely associated with perceptual input during a traumatic event. It has been proposed, therefore, that intrusive memories arise when cue-driven activation of trauma-related representations in associative networks is poorly controlled, that is, when representations are very readily activated (Ehlers & Clark, 2000; Michael & Ehlers, 2007). Related to that, previous research indicated that, in individuals showing prefrontal-posterior de-coupling during the processing of emotionally laden sensory input, emotion-congruent representations were more readily activated. For instance, individuals showing greater decreases of prefrontal-posterior EEG beta coherence during the observation of other people's expressions of cheerfulness rated cartoons as being more funny than individuals in whom prefrontal-posterior coherence had decreased to a lesser extent or had increased. On the other hand, a decrease of prefrontal-posterior EEG coherence during the exposure to negative affect expressions predicted greater difficulties to judge one's amusement, probably due to poorer inhibition of nascent negative feelings evoked by sympathy with the victims in the jokes (Papousek et al., 2013). Similarly, prefrontal-posterior functional de-coupling during perceptual processing may be linked to the ready activation of respective memory representations, which in turn is thought to be related to implicit memory encoding (Brewin, 2014; Michael & Ehlers, 2007).

As was expected, only EEG coherences in the right, but not in the left, hemisphere predicted the occurrence of intrusive memories. Previous studies have suggested a particular importance of prefrontal-posterior coupling in the right hemisphere for explicit versus implicit memory encoding (Rose et al., 2010; Wessel et al., 2012), although there is some inconsistency across studies (McIntosh et al., 1999). The use of exclusively visual material, which may be preferentially processed in the right hemisphere, may also play a role in this context. Viewed from the perspective of the affective research tradition, the present results may be in line with the predominant role of the right hemisphere in emotion processing, in particular in terms of the intensity of emotional arousal (Gainotti, 2000; Hagemann, Hewig, Naumann, Seifert, & Bartussek, 2005; Papousek, Schulter & Lang, 2009). Most studies examining changes of functional coupling in affective contexts have reported stronger associations with EEG coherence changes in the right than in the left hemisphere (Papousek et al., 2013; Reiser et al., 2012; Schellberg et al., 1990; but see Miskovic & Schmidt, 2010).

In contrast to intrusive memories occurring immediately after viewing the film and intrusive memories occurring over the week as assessed with the intrusion scale of the IES-R, EEG coherence changes during viewing the film did not predict the number of intrusions noted in the daily diary. Several reasons may account for this difference. The mean number of intrusive memories noted in the diary was somewhat lower than in previous studies (Holmes et al., 2009, 2010). The strong dependence on the conscientiousness and compliance of the participants gives rise to problems in the diary assessment (Bolger, Davis, & Rafaeli, 2003). It cannot be ruled out that at least some of the participants had not fully complied with the requirement to keep the diary daily, while the tasks administered in the lab would not have had this limitation. Compliance ratings should be added in future studies.

When considering the present findings, one has to keep in mind that, compared to experiencing or witnessing real traumatic events, the potential of the used film to produce intrusive memories was only relatively weak. However, it is all the more remarkable that plausible associations between individual differences in the functional coupling of prefrontal and posterior cortices and the development of intrusive memories were shown, not only immediately after exposure to the traumatic content, but also during the subsequent week. At the same time, the findings suggest that viewing film content of the kind that is shown in television programs such as reality or news programs can have some impact on the viewers (see also Breslau et al., 2010; Schlenger et al., 2002; Schuster et al., 2001), and indeed traumatic film footage viewed in work situations may even lead to PTSD symptoms (American Psychiatric Association, 2013). Thus, in addition to providing indications of processes that may be relevant to the development of clinical symptoms (see also Holmes & Bourne, 2008), the present experimental study may show neurological processes that are relevant to intrusive memories in everyday life and in the subclinical domain, which can also be burdensome (Krans et al., 2009).

A limitation of the present study is that implicit (rather than explicit) memory encoding of the contents that later popped up as intrusions could not be verified empirically. In that respect, we have to rely on the theoretical background and empirical evidence outlined in the introduction (Brewin, 2001; Brewin et al., 1996; Cohen et al., 2012; Dehaene & Changeux, 2011; Dehaene et al., 2006; Holmes et al., 2004; McIntosh et al., 1999; Rose et al., 2010; Wessel et al., 2012). The study design may have introduced some error variance, because the film scenes may have been more prone to eye movements than the static image used for obtaining the reference data. However, it is important to consider that the research question of the present study did not refer to the main effect of condition on EEG coherence but to individual differences in state-dependent coherence changes, specifically to their relation to the occurrence of intrusive memories. Thus, a greater amount of eye movements during the film than during the reference period could only have produced spurious findings, if the difference in eye movements (or any other response to physical differences between the stimuli) had been systematically related to the dependent variables (i.e., individual differences in the occurrence of intrusive memories). Specifically, this would mean that participants in whom prefrontal-posterior coherence decreased would have had to show reduced eye movements during watching the film compared to the neutral display, and at the same time be more prone to intrusions. This seems very unlikely. Similarly, this applies to the different length of the recording periods, which could also only have influenced the findings, if there had been a systematic covariation of the difference between the number of artifact-free epochs in the two recording periods with the coherence changes as well as with the dependent variables. No such correlations were present. A potential limitation of the study is that volume conduction artifacts cannot be completely excluded when exploring EEG coherences. The absence of correlations between diagonal (left–right and right–left) prefrontal-posterior coherences and the dependent variables provides some indication that the findings were not explained by a strong source producing spurious coherences because it had influenced the signals at anterior as well as at posterior electrodes. However, more research, preferably also using other methods to test for individual differences in intrahemispheric coupling, will be needed to more exactly clarify the brain mechanisms underlying the present findings (e.g., Nolte et al., 2004). Further, the explanatory power of the findings is limited by the modest size of the effects. Large effects are generally not to be expected in brain research, because all psychological processes always involve several brain structures and mechanisms. Moreover, the potential of the used procedure to evoke intrusive memories was only moderate. Of course, ethical considerations play a major role in this context because studying brain processes during the experience or witnessing of real trauma is clearly impossible.

In conclusion, merging cognitive and emotional brain research (Isaac & Bayley, 2012), the findings of the present study illuminate novel brain mechanisms involved in the encoding of information in ways that make intrusive memories more likely. In contrast to research on hypoactivation of brain areas and its relation to deficits in cognitive and affective functioning, the importance of functional connectivity changes between cortical units to personality and psychopathology has been relatively sparsely examined to date. The present study adds to the evidence that investigating interindividual differences in corticocortical communication may indeed deliver us insight into mechanisms not fully understood so far, such as the development of intrusive memories. Given the distress they can cause, the understanding of such underlying mechanisms is crucial.

## Footnotes

1 Additional data were obtained for purposes related to other, nonoverlapping research questions. These include genetic data and EEG asymmetries, reported for a larger sample (first experimental session only) in Papousek et al. (2013).

2 Analyses in other frequency bands did not reach the significance level.

3 Analyses in other frequency bands did not reach the significance level.
